# A 3C-SiC-on-Insulator-Based Integrated Photonic Platform Using an Anodic Bonding Process with Glass Substrates

**DOI:** 10.3390/mi14020399

**Published:** 2023-02-06

**Authors:** Jiayang Li, Andrew W. Poon

**Affiliations:** Photonic Device Laboratory, Department of Electronic and Computer Engineering, The Hong Kong University of Science and Technology, Clear Water Bay, Hong Kong

**Keywords:** 3C-SiC, SiC photonics, integrated photonics, anodic bonding, microring resonators

## Abstract

Various crystalline silicon carbide (SiC) polytypes are emerging as promising photonic materials due to their wide bandgap energies and nonlinear optical properties. However, their wafer forms cannot readily provide a refractive index contrast for optical confinement in the SiC layer, which makes it difficult to realize a SiC-based integrated photonic platform. In this paper, we demonstrate a 3C-SiC-on-insulator (3C-SiCoI)-based integrated photonic platform by transferring the epitaxial 3C-SiC layer from a silicon die to a borosilicate glass substrate using anodic bonding. By fine-tuning the fabrication process, we demonstrated nearly 100% area transferring die-to-wafer bonding. We fabricated waveguide-coupled microring resonators using sulfur hexafluoride (SF_6_)-based dry etching and demonstrated a moderate loaded quality (Q) factor of 1.4 × 10^5^. We experimentally excluded the existence of the photorefractive effect in this platform at sub-milliwatt on-chip input optical power levels. This 3C-SiCoI platform is promising for applications, including large-scale integration of linear, nonlinear and quantum photonics.

## 1. Introduction

Beyond the recent interest in the semiconductor microelectronics industry for power devices, various silicon carbide (SiC) polytypes, including 4H-, 6H- and 3C-SiC, are emerging as attractive photonic materials. SiC features a wide bandgap energy from 2.3 eV (3C-) to 3.2 eV (4H-) [1], which offers a wide transparent window and excludes the nonlinear two-photon absorption for light in the near-infrared telecommunications bands. As a result of the non-centrosymmetric lattice structures of SiC, these materials feature non-vanishing second-order nonlinear optical susceptibilities, which is promising for nonlinear and quantum photonics. Moreover, the sulfur hexafluoride (SF_6_)-based dry etching process of SiC and the resulting Si-/C- etching by-products [2] offer better compatibility with the complementary metal–oxide–semiconductor (CMOS) process compared with other nonlinear photonic materials, including LiNbO_3_ and AlGaAs. SiC also features a moderate refractive index (~2.6) at the near-infrared (NIR) wavelengths. This enables tight optical confinement within the SiC waveguide core against low-refractive-index cladding materials, including silicon dioxide (SiO_2_). SiC integrated photonic devices potentially have a compact footprint. However, SiC thin-films with a refractive index contrast are not readily available from commercial SiC wafers. Typically, 4H-/6H-SiC thin-films are grown into bulk substrates, whereas 3C-SiC thin-films are epitaxially grown on Si substrates, which have a larger refractive index than the SiC layer, and are thus not suitable for integrated photonic applications. Those hard SiC films cannot be wet-transferred as soft materials, for example, graphene, can [3]. Hence, methods to realize SiC-on-insulator (SiCoI)-based integrated photonic platforms have drawn significant research interest recently.

For bulk wafers, researchers demonstrated 4H-/6H-SiCoI platforms by first bonding the bulk SiC wafer on a Si wafer with a thermal SiO_2_ optical insulating layer through a molecular bonding process [4,5,6,7]. This is a highly demanding process requiring strict surface cleanness and a low surface roughness of <1 nm root-mean-square (RMS) [8]. The process typically adopts industrial-level wafer grinding and chemical mechanical polishing (CMP) equipment to remove and polish away the bulk of the SiC wafer, followed by dry etching to reach the target SiC film thickness [4,5,6]. These processes waste the bulk of the SiC wafer and cause a large wafer-scale thickness non-uniformity [4]. Despite the aforementioned issues, researchers demonstrated a high loaded Q factor of 9.7 × 10^5^ and third-order optical parametric oscillation based on this platform [5]. They also demonstrated second-harmonic generation from a microring resonator with loaded Q values of 8 × 10^4^ and 2 × 10^4^ at 1550 nm and 780 nm, respectively [6]. An alternative method to control SiC film thickness is using the smart-cut method [7]. Researchers demonstrated a loaded Q value of 7.4 × 10^4^ from a microring resonator based on this platform. This technique can result in a well-controlled film thickness after bonding. However, it imposes a large implantation dose of H ions into the 4H-SiC wafer, which is demanding for the equipment. The damages caused by ion bombardment potentially degrade the SiC layer [9].

Growing crystalline thin-films on Si wafers potentially offers a cost-effective and wafer-level scalability for mass manufacturing [10,11,12,13]. Among all the SiC polytypes, 3C-SiC is the only cubic form, and can be epitaxially grown on Si substrates [10]. After bonding the 3C-SiC-on-Si wafer on another optical insulating wafer through a molecular bonding process, researchers can readily remove the Si substrate via dry and wet etching because the 3C-SiC film can serve as an etch stop layer [14,15,16]. The exposed 3C-SiC surface is the original SiC/Si interface, which has a poor crystal quality and is often removed using the following dry etching process. Researchers demonstrated intrinsic Q values of 1.42 × 10^5^ and 2.42 × 10^5^ from a microring and a microdisk resonator based on this platform, respectively [14,15]. Researchers also demonstrated an electro-optic modulator of approximately 1550 nm with a 3dB bandwidth of 7.1 GHz using a microring resonator with an intrinsic Q value of 8.9 × 10^4^ [16]. One way to avoid the highly demanding molecular bonding process is to under-cut the Si substrate to form suspended 3C-SiC resonators and waveguides [17,18,19]. This approach is less mechanically stable and the original low-quality SiC surface is not removed. The recently reported intrinsic Q value of microrings from this platform is 4.1 × 10^4^ [19]. Researchers demonstrated a loaded Q value of 1.4 × 10^5^ from a microring resonator using amorphous SiC deposited on a thermal oxide layer [20]. However, the amorphous films do not feature second-order nonlinear optical susceptibilities in bulk. 

In this study, we adopted and optimized the anodic bonding process instead of the molecular bonding process to enable a reliable and fast method to transfer the 3C-SiC film onto the optical insulating layer. The anodic bonding technique has been developed and leveraged in wafer packaging and in micro-electro-mechanical systems [21,22,23], which bonds Si-based substrates and borosilicate glass substrates. This concept fits our requirements of demonstrating a 3C-SiCoI platform for integrated photonics. After process tuning, we demonstrated nearly 100% 3C-SiC film area transferring from the Si wafer to the glass substrate in a die-to-wafer anodic bonding process. The process took only 68 s, which is significantly faster than the conventional molecular bonding process [8]. To our knowledge, this is the first time that the anodic bonding process has been applied to integrated SiC photonics. We etched the devices using a SF_6_-based deep reactive ion etching (DRIE) recipe and demonstrated a loaded Q value of up to 1.4 × 10^5^, approximately 1550 nm wavelengths from a waveguide-coupled microring resonator, which is comparable with the state-of-the-art [14]. We tuned the input optical power into the device and experimentally excluded the photorefractive effect under a sub-milliwatt on-chip input power level. The absence of the photorefractive effect can make nonlinear photonic devices more controllable compared with those realized in other material platforms, for example, LiNbO_3_ [24]. 

## 2. Platform Development

**Figure 1:**
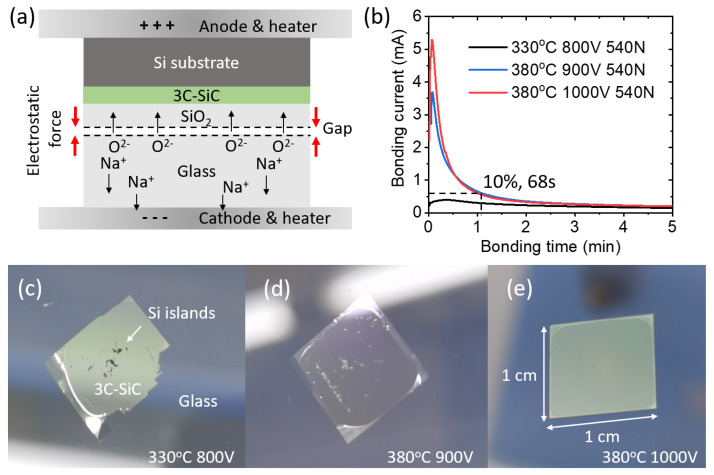
(**a**) An illustration of the principle of the anodic bonding process applied to 3C-SiC on a glass substrate. (**b**) Measured total bonding currents flowing through four 1 × 1 cm^2^ 3C-SiC dies during the bonding process under different conditions. (**c**–**e**) Successfully transferred 3C-SiC films on glass substrates after Si substrate removal under different process conditions. The remaining Si islands could be readily removed by wet-etching using mixed acid solutions.

The key step in realizing our 3C-SiCoI platform was anodic bonding. Figure 1a schematically illustrates the bonding mechanism. The Si substrate and the glass substrate were first placed into contact in the bonder, then the whole system was heated to 300–500 °C in a vacuum. Under such an elevated temperature, the borosilicate glass substrate containing movable sodium and oxygen ions is regarded as a good conductor [21]. This means that after applying a voltage across the Si-glass pair, a significant portion of the field was applied within any existing non-conducting gap spacing between the sample pair. A resulting large electrostatic attractive force drew the pair into intimate contact, then the movable oxygen ions could be attracted into the Si substrate side and form a permanent bonding [23]. The field-induced migration of sodium and oxygen ions generated a bonding current across the two electrodes. Bonding was complete when all the movable ions around the interface were depleted and the bonding current decreased to the background level as a result. Based on these mechanisms, the surface roughness requirement is reported as RMS < 20 nm [25], which is significantly relaxed compared with the RMS < 1 nm required by the molecular bonding process.

We developed the platform in our university’s clean room. We diced the commercial 4″ epitaxial 3C-SiC-on-Si wafers (NOVASiC) into 1 × 1 cm^2^ dies for die-to-wafer process optimization. The 3C-SiC film thickness was 1.5 μm with a CMP polishing from the company. At this stage, we characterized the 3C-SiC film with an ellipsometer and fitted the refractive index in the transparent window of this material using a Cauchy model. We obtained the A, B coefficients of 2.553 and 0.0353, respectively. These fitted values allowed us to extract the film thickness during the fabrication process and to calculate the waveguide modal information. We chose 4” thermo-resist borosilicate glass substrates (Borofloat 33) as the target substrate. As these glass wafers contain impurities, we deposited a layer of plasma-enhanced chemical vapor deposition (PECVD) SiO_2_ with a thickness of 500 nm onto the 3C-SiC sample before bonding as a high-quality under-cladding layer. As an insulating material, the addition of a SiO_2_ layer required a higher bonding voltage for optimization. After cleaning, using 120 °C H_2_SO_4_ (98%):H_2_O_2_ (30%) = 10:1 solution on both samples, we placed four 3C-SiC dies onto the glass substrate and into a wafer bonder (Karl Suss SB6). Figure 1b shows the recorded bonding current during the short bonding process under different conditions. We observed that under a lower temperature of 330 °C, the bonding current was small due to the low mobility of the ions inside the glass wafer. After raising the temperature to 380 °C, the bonding current increased significantly. Raising the bonding voltage from 900 to 1000 V also boosted the bonding current, which indicates that more oxygen ions crossed the interface, suggesting a better bonding. We noticed that the bonding current dropped to 10% of the peak after only 68 s of processing, indicating the bonding process was approaching completion. This was much faster than the conventional molecular bonding process adopted in other works, which takes hours of annealing for better results. The recorded force of 540 N originated from the self-weight of the electrode of the bonder to hold the samples inside. Figure 1c–e show the improvements in the transferred 3C-SiC film area. Under a temperature of 380 °C and a voltage of 1000 V, which are typical operating conditions for wafer bonders, we obtained an almost 100% 3C-SiC film area transferring ratio onto the glass substrate in only 68 s. This process could be readily exploited for a larger-scale wafer-to-wafer bonding, as a larger area only requires more ions to flow across, which may require a longer completion time.

**Figure 2:**
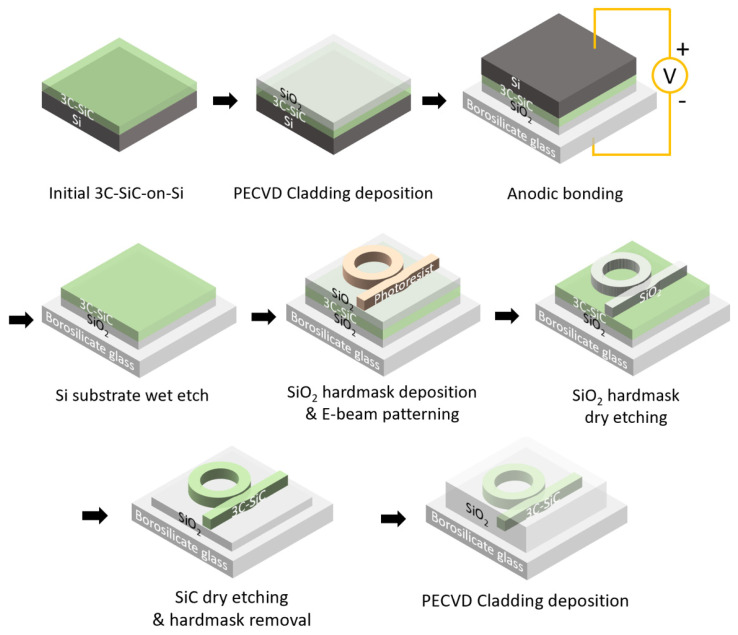
Schematics of the key steps of the fabrication process.

Figure 2 schematically illustrates the fabrication process flow. After the anodic bonding process, we used a 25% tetramethylazanium hydroxide solution heated to 80 °C for wet etching, which only removed the Si substrate. We removed the remaining Si islands using the mixed acid solution (nitric/hydrofluoric acid/acetic acid) for a short time before etching significantly into the glass substrate. We developed a fluorine-based DRIE recipe with a gas flow of SF_6_:O_2_ = 5:1 to dry etch the 3C-SiC layer. We thinned the film to a thickness of 800 nm to remove the 3C-SiC portion that contained significant epitaxial defects. Then we deposited a layer of PECVD SiO_2_ as a hardmask layer. We used electron-beam (E-beam) lithography to pattern the photoresist on the hardmask. We transferred the patterns to the hardmask using a fluorine-based inductively coupled plasma reactive ion-etching recipe. Afterwards, we transferred the patterns onto the SiC layer using the same recipe from the SiC thinning step. We removed 700 nm of the SiC and left a slab thickness of 100 nm for mechanical stability of the bonded film. We noted an etching selectivity of 3C-SiC:SiO_2_ = 1.45. Afterward, we used buffered oxide etch (BOE) for SiO_2_ wet etching in the hardmask removal step. As the SiC slab layer protected the underneath SiO_2_ cladding layer, we dipped the sample in BOE for 5 minutes, which was long enough to completely remove the remaining hardmask and to expose the SiC waveguide structures. Finally, we deposited a layer of PECVD SiO_2_ with a thickness of 500 nm onto the chip to protect the devices. We cleaved the chip into columns to open the waveguide facets for experiments.

**Figure 3:**
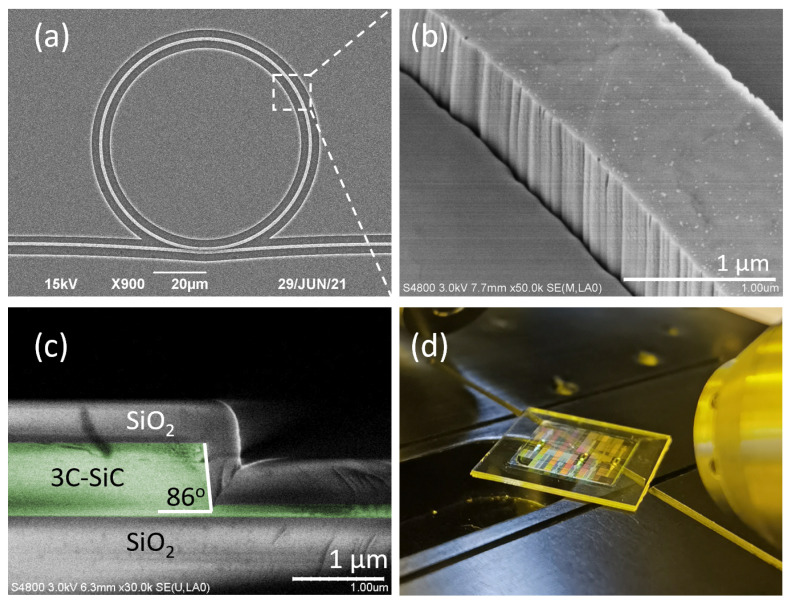
(**a**) A scanning-electron micrograph (top-view) of the fabricated waveguide-coupled microring resonator. (**b**) A zoom-in view showing the waveguide sidewall roughness. (**c**) A cross-sectional view of the fabricated waveguide facet, featuring a sidewall slope of 86°. (**d**) A picture of the 3C-SiCoI chip.

Figure 3a shows a fabricated waveguide-coupled microring resonator. Figure 3b shows a zoom-in view illustrating the sidewall quality of the waveguide. The significant sidewall roughness indicates that our dry etching recipe required further optimizations. The sidewall roughness added random perturbations to the waveguide cross-section along the propagation direction and caused excessive scattering losses [26]. Figure 1c shows a cross-sectional view of the fabricated waveguides, showing a sidewall slope of ~86°. Such a steepness was desirable for opening a waveguide-to-microring coupling gap spacing of 200 nm. Figure 1d shows the final integrated 3C-SiCoI chip after all the fabrication steps.

## 3. Experimental Results

**Figure 4:**
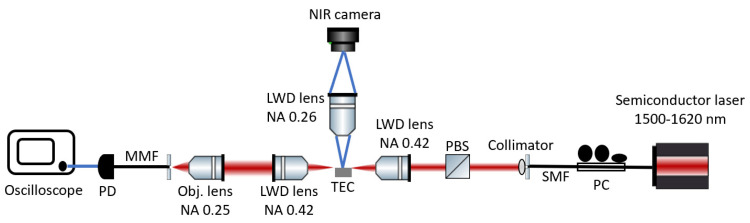
Schematic of the experimental setup.

Figure 4 shows a schematic of the experimental setup for optical transmission characterizations of the fabricated devices. We used a wavelength-tunable (1500–1620 nm) continuous-wave (CW) laser. We used a single-mode fiber (SMF)-based polarization controller (PC) and a polarization beam splitter (PBS) to control the input light polarizations. We mounted the chip on a feedback-controlled thermal-electric controller (TEC) to maintain a stable chip temperature of 22 °C during measurements. We used a pair of long-working-distance (LWD) objective lenses with a numerical aperture (NA) of 0.42 to conduct butt coupling with the sample. We used an NIR camera to monitor the out-of-plane scattered light on top to optimize the coupling. We coupled the transmitted light into a multi-mode fiber (MMF) and detected the spectrum using a photodetector (PD) and an oscilloscope. 

**Figure 5:**
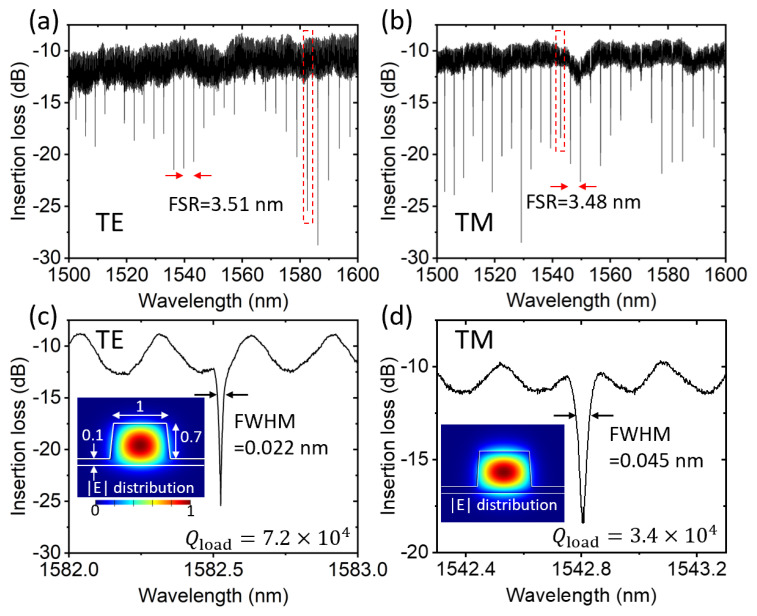
Measured transmission spectra normalized to the lens-to-lens coupling in (**a**) TE and (**b**) TM polarizations of a microring resonator. (**c**,**d**) Zoom-in views of the resonances within the red dashed-line boxes of (**a**) and (**b**), respectively. Insets: Numerically simulated TE- and TM-polarized mode-field amplitude distributions with the waveguide dimensions labeled in μm units.

Figure 5 shows the measured transmission spectra normalized to the lens-to-lens transmission from a circular microring resonator device, with a waveguide width of 1 μm and a microring radius of 40 μm. The coupling bus-waveguide had a width of 1 μm, a coupling gap spacing of ~200 nm and an interaction length or a wrapping distance of 14 μm. Figure 5a,b show the measured spectra with the transverse electric (TE) and transverse-magnetic (TM)-polarized input light, respectively. The free-spectral range (FSR) around 1550 nm was 3.51 nm for TE-mode and 3.48 nm for TM-mode. Based on the relationship:

(1) $$FSR=\lambda^{2}/n_{g}L,$$

where *λ*, *n_g_* and *L* are the central resonance wavelength, the effective modal group refractive index and the resonator circumference, respectively, we calculated an $n_{g,\mathrm{TE}}=2.73$ and $n_{g,\mathrm{TM}}=2.75$, which were close to and featuring the same trend with the numerically simulated values of $n_{g,\mathrm{TE},\mathrm{simu}}=2.74$ and $n_{g,\mathrm{TM},\mathrm{simu}}=2.78$. Figure 5c,d show the zoom-in views of particular TE- and TM-resonances with the full-width at half-maximum (FWHM) labeled, respectively. The insets show the numerically calculated field-amplitude distributions of these modes. The loaded Q value was calculated as follows:

(2) Q=λ/FWHM 

The TE-modes, with extinction ratios of ~10 dB, typically featured loaded Q values at the high-end of 10^4^, whereas the TM-modes had smaller loaded Q values. The Fabry–Pérot (FP)-like modulation background was possibly caused by the back-reflections of the two cleaved waveguide facets, with the modulation period of ~0.3 nm being consistent with the bus-waveguide length of 1.5 mm.

**Figure 6:**
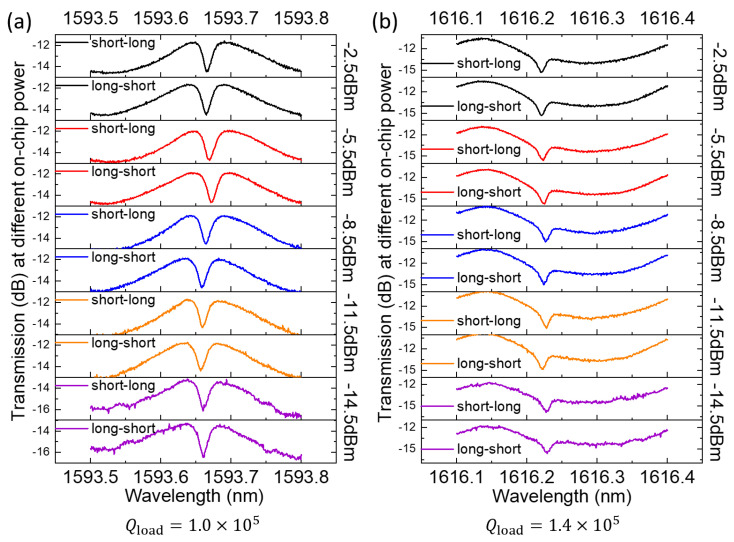
Measured transmission spectra of two different TE-polarized resonances under different on-chip input powers (−14.5 dBm~−2.5 dBm) with different wavelength-scanning directions (i.e., from short (long) wavelength to long (short) wavelength) with loaded Q values of (**a**) 1.0 × 10^5^ and (**b**) 1.4 × 10^5^, respectively.

We observed a loaded Q value of TE-modes up to 1.4 × 10^5^ at ~1616 nm from another microring resonator, which featured an interaction length of 7 μm, with all other dimensions the same as the one discussed above. We did not include the zoom-out view of its spectrum because the weaker coupling resulted in much shallower resonance dips, which were comparable to the background FP modulation. Instead, we performed input power and wavelength-scanning direction dependence measurements on this resonator using these high Q resonances to examine the existence of the photorefractive effect. Figure 6a,b show a myriad of transmission spectra of two resonances under different sub-milliwatt on-chip input powers under two wavelength-scanning directions. The resonance shapes remained essentially the same under various conditions, which indicates that there were no significant photorefractive effects compared with the literature results from an LiNbO_3_ resonator under similar Q values and input powers [24].

Table 1 summarizes the reported Q values from SiC and other common semiconductor microring resonators using different fabrication schemes. Our demonstrated Q values are comparable to those in the state-of-the-art of 3C-SiC. However, we noted that the Q values of our 3C-SiC platform were still several times lower than the state-of-the-art of the 4H-SiC platform. We attributed this to the rough waveguide sidewalls caused by our un-optimized dry etching process.

## 4. Conclusions

We demonstrated a 3C-SiCoI integrated photonic platform using an optimized die-to-wafer anodic bonding process. This process featured a minute-scale processing time, a relaxed surface roughness requirement for bonding and a nearly 100% 3C-SiC film area transferring ratio onto the glass wafer. Applying a SF_6_-based SiC etching recipe, we demonstrated a loaded Q factor of up to 1.4 × 10^5^ from a waveguide-coupled microring resonator, which is comparable to the state-of-the-art using this material. We experimentally demonstrated that there was no significant photorefractive effect under a sub-milliwatt on-chip input power level. It was possible to increase the microring Q factors by optimizing the SiC etching recipe to minimize the waveguide sidewall roughness. Our work demonstrated a promising 3C-SiCoI platform for linear, nonlinear and quantum SiC photonic circuits.

## Figures and Tables

**Table 1:** A summary of different platforms for integrated photonics.

Platform	Key Fabrication Steps	Device Parameters (Thickness, Width ^1^, Radius)	Reported Loaded Q’s
Silicon-on-insulator [27]	Smart-cut and Molecular bonding	220 nm, 2 μm, 20 μm	1.14 × 10^6^
Silicon nitride [28]	Deposition	730 nm, 10 μm, 369 μm	6.7 × 10^7 3^
LiNbO_3_ [29]	Smart-cut and Molecular bonding	600 nm, 2.4 μm, 80 μm	5 × 10^6^
AlGaAs-on-insulator [30]	Molecular bonding	400 nm, 0.69 μm, 13.91 μm	1.24 × 10^6^
Amorphous SiC [20]	Deposition	350 nm, 0.8 μm, 50 μm	1.4 × 10^5^
4H-SiCoI [4]	Molecular bonding and Wafer polishing	530 nm, 3 μm, 100 μm ^2^	9.7 × 10^5^
4H-SiCoI [6]	Smart-cut and Molecular bonding	500 nm, 0.75 μm, 16.5 μm ^2^	7.4 × 10^4^
3C-SiCoI [14,15]	Molecular bonding	800 nm, 1.7 μm, 40 μm 500 nm, −60 μm (disk)	1.42 × 10^5 3^ 2.42 × 10^5 3^
3C-SiC suspended [19]	Si undercut	450 nm, 0.58 μm, 40 μm	4.1 × 10^4 3^
This work, 3C-SiCoI	Anodic bonding	800 nm, 1 μm, 40 μm	1.4 × 10^5^

^1^ We adopted the top widths for comparison, as all the SiC waveguide sidewalls had certain slopes; ^2^ The authors did not specify the definition of the widths; ^3^ Only intrinsic Q values are reported.
